# Acetonyltriphenyl­phospho­nium nitrate

**DOI:** 10.1107/S1600536813002110

**Published:** 2013-01-31

**Authors:** Tidiane Diop, Libasse Diop, Monika Kučeráková, Michal Dušek

**Affiliations:** a Laboratoire de Chimie Minerale et Analytique, Departement de Chimie, Faculté des Sciences et Techniques, Université Cheikh Anta Diop, Dakar, Senegal; b Institute of Physics ASCR, v.v.i., Na Slovance 2, 182 21 Praha 8, Czech Republic

## Abstract

Crystals of the title salt, C_21_H_20_OP^+^·NO_3_^−^, are composed of acetonyltriphenyl­phospho­nium cations and nitrate anions that mainly inter­act through electrostatic forces. The P atom in the cation has a slightly distorted tetra­hedral environment, with C—P—C angles ranging from 104.79 (7) to 112.59 (6)°. The sum of O—N—O angles of the nitrate anion is 359.99°, reflecting its trigonal–planar character. C—H⋯O hydrogen bonds help to consolidate the crystal packing.

## Related literature

For crystal structures containing triphenyl­phospho­nium moieties, see: van der Sluis & Spek (1990 ▶); Boys *et al.* (1995 ▶); Zhang *et al.* (2004 ▶); Evans (2010 ▶); Kavitha *et al.* (2012 ▶).
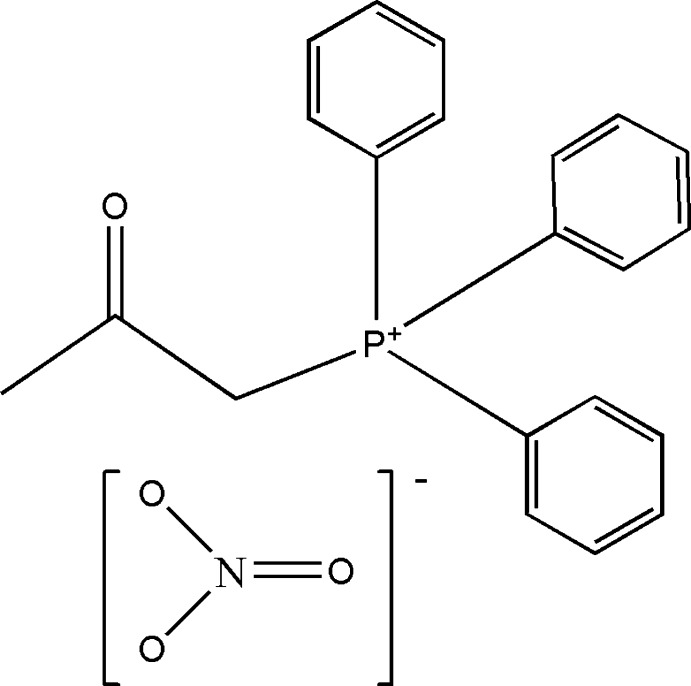


## Experimental

### 

#### Crystal data

C_21_H_20_OP^+^·NO_3_^−^
*M* *_r_* = 381.4
Monoclinic, 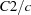

*a* = 14.0928 (5) Å
*b* = 12.6455 (3) Å
*c* = 21.2684 (6) Å
β = 90.667 (2)°
*V* = 3790.00 (19) Å^3^
*Z* = 8
Cu *K*α radiation
μ = 1.51 mm^−1^
*T* = 120 K
0.19 × 0.18 × 0.12 mm

#### Data collection

Agilent Xcalibur diffractometer
Absorption correction: multi-scan (*CrysAlis PRO*; Agilent, 2012) ▶ *T*_min_ = 0.271, *T*_max_ = 1
22035 measured reflections
3389 independent reflections
2969 reflections with *I* > 3σ(*I*)
*R*_int_ = 0.040

#### Refinement

*R*[*F* ^2^ > 3σ(*F* ^2^)] = 0.033
*wR*(*F* ^2^) = 0.094
*S* = 1.63
3389 reflections
250 parameters
2 restraints
H atoms treated by a mixture of independent and constrained refinement
Δρ_max_ = 0.27 e Å^−3^
Δρ_min_ = −0.24 e Å^−3^

### 

Data collection: *CrysAlis PRO* (Agilent, 2012 ▶); cell refinement: *CrysAlis PRO*; data reduction: *CrysAlis PRO*; program(s) used to solve structure: *SUPERFLIP* (Palatinus & Chapuis, 2007 ▶); program(s) used to refine structure: *JANA2006* (Petříček *et al.*, 2006 ▶); molecular graphics: *DIAMOND* (Brandenburg & Putz, 2005 ▶); software used to prepare material for publication: *JANA2006*.

## Supplementary Material

Crystal structure: contains datablock(s) global, I. DOI: 10.1107/S1600536813002110/wm2713sup1.cif

Structure factors: contains datablock(s) I. DOI: 10.1107/S1600536813002110/wm2713Isup2.hkl

Supplementary material file. DOI: 10.1107/S1600536813002110/wm2713Isup3.cml

Additional supplementary materials:  crystallographic information; 3D view; checkCIF report

## Figures and Tables

**Table 1:** Hydrogen-bond geometry (Å, °)

*D*—H⋯*A*	*D*—H	H⋯*A*	*D*⋯*A*	*D*—H⋯*A*
C5—H1*c*5⋯O3^i^	0.960 (13)	2.252 (13)	3.2053 (18)	172.1 (12)
C5—H3*c*5⋯O2^ii^	0.960 (13)	2.403 (12)	3.1936 (18)	139.4 (11)
C7—H1*c*7⋯O3^i^	0.96	2.49	3.4365 (19)	167.90
C8—H1*c*8⋯O3	0.96	2.50	3.177 (2)	127.75
C10—H1*c*10⋯O2^ii^	0.96	2.49	3.3706 (19)	152.50
C15—H1*c*15⋯O1	0.96	2.36	3.1780 (19)	142.99

Symmetry codes: (i) 
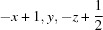
; (ii) 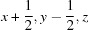.

**Fig. 1.**
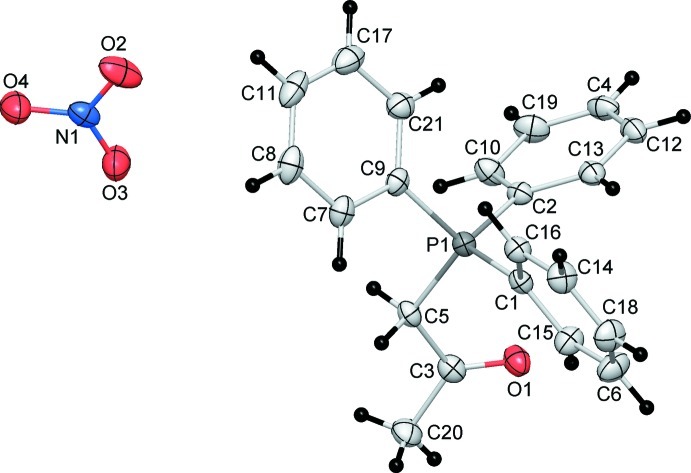
The molecular entities of the title compound with displacement ellipsoids drawn at the 50% probability level.

**Fig. 2.**
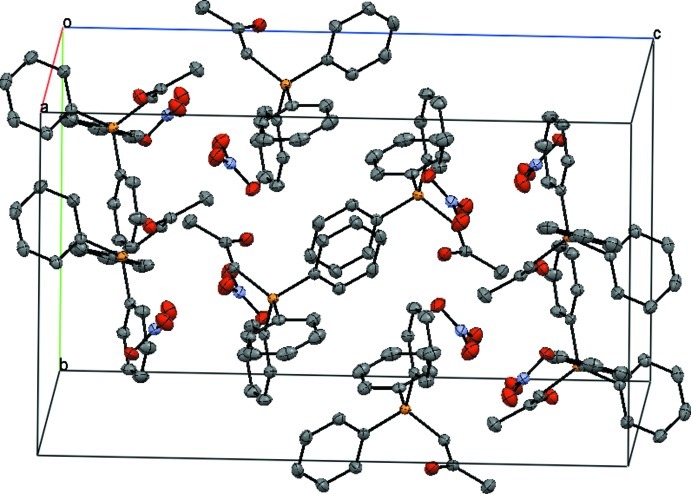
The crystal packing of the title compound.

## supplementary crystallographic information

### Comment

Phosphonium salts [P*R*_4_^+^, *R* = alkyl or aryl] are widely used as large cations to stabilize a variety of anonic species (Zhang *et al.*, 2004; van der Sluis & Spek, 1990; Evans, 2010).

The title compound crystallizes with one phosphonium cation, C_21_H_20_OP^+^, and one nitrate anion in the asymmetric unit (Fig. 1). The P—C bond lengths within the cation [1.7947 (13), 1.7984 (14), 1.7992 (15) and 1.8024 (13) Å] are similar than those reported for related phosphonium salts like [1-(ethoxycarbonyl)-1-cyclopentyl]triphenylphosphonium bromide (Boys *et al.*, 1995), or [3-(iodoacetamido)propyl]triphenylphosphonium tetraphenylborate (Evans, 2010) indicating that the presence of the acetonyl moiety has a negligible effect on the geometrical parameters. The C—P—C angles (range 104.79 (7) to 112.59 (6) °) indicate a slight angular distortion. The sum of the O—N—O angles [120.84 (14), 120.16 (14) and 118.99 (12) °] of the nitrate anion is 359.99°, reflecting its trigonal-planar geometry. Between the C_21_H_20_OP^+^ cations and the NO_3_^-^ anions, the interactions are mainly of electrostatic nature. Such forces are also respondible for related salts like (3-chloropropyl)triphenylphosphonium bromide (Kavitha, 2012). The packing of the structure is shown in Fig. 2. Weak C—H···O hydrogen bonds (Table 1) help to consolidate the crystal packing.

### Experimental

All chemicals were purchased from Aldrich (Germany) and used without any further purification. Colourless crystals of the title compound, C_21_H_20_OP^+^^.^NO_3_^-^, have been obtained by the addition of a solution of Pb(NO_3_)_2_ (0.46 g,1.4 mmol) in water to a solution of CH_3_COCH_2_P(C_6_H_5_)_3_Cl (0.5 g, 1.4 mmol) in water. The precipitated PbCl_2_ was filtered off. Regular crystals were grown after slow solvent evaporation within few days.

### Refinement

Hydrogen atoms, except H1c5 and H3c5, were kept in the geometrically correct positions with a C—H distance of 0.96 A. The tetrahedron around C5 contains phosphorus at one apex, therefore positions of H1C5 and H3C5 were refined with a C—H distance restraint of 0.96 Å (σ of the restraint 0.001). Isotropic temperature factors of all hydrogen atoms were calculated from *U*_eq_ of the corresponding parent atom multiplied by 1.2.

### Crystal data

**Table:**

C_21_H_20_OP^+^·NO_3_^−^	*F*(000) = 1600
*M**_r_* = 381.4	*D*_x_ = 1.336 Mg m^−^^3^
Monoclinic, *C*2/*c*	Cu *K*α radiation, λ = 1.5418 Å
Hall symbol: -C 2yc	Cell parameters from 12744 reflections
*a* = 14.0928 (5) Å	θ = 4.2–67.0°
*b* = 12.6455 (3) Å	µ = 1.51 mm^−^^1^
*c* = 21.2684 (6) Å	*T* = 120 K
β = 90.667 (2)°	Polygon, colourless
*V* = 3790.00 (19) Å^3^	0.19 × 0.18 × 0.12 mm
*Z* = 8	

### Data collection

**Table:**

Agilent Xcalibur diffractometer	3389 independent reflections
Radiation source: Enhance Ultra (Cu) X-ray Source	2969 reflections with *I* > 3σ(*I*)
Mirror monochromator	*R*_int_ = 0.040
Detector resolution: 10.3784 pixels mm^-1^	θ_max_ = 67.1°, θ_min_ = 4.2°
ω scans	*h* = −16→16
Absorption correction: multi-scan (*CrysAlis PRO*; Agilent, 2012)	*k* = −15→15
*T*_min_ = 0.271, *T*_max_ = 1	*l* = −25→24
22035 measured reflections	

### Refinement

**Table:**

Refinement on *F*^2^	74 constraints
*R*[*F*^2^ > 2σ(*F*^2^)] = 0.033	H atoms treated by a mixture of independent and constrained refinement
*wR*(*F*^2^) = 0.094	Weighting scheme based on measured s.u.'s *w* = 1/(σ^2^(*I*) + 0.0016*I*^2^)
*S* = 1.63	(Δ/σ)_max_ = 0.015
3389 reflections	Δρ_max_ = 0.27 e Å^−^^3^
250 parameters	Δρ_min_ = −0.24 e Å^−^^3^
2 restraints	

### Special details

**Table:**

Refinement. The refinement was carried out against all reflections. The conventional *R*-factor is always based on *F*. The goodness of fit as well as the weighted *R*-factor are based on *F* and *F*^2^ for refinement carried out on *F* and *F*^2^, respectively. The threshold expression is used only for calculating *R*-factors *etc*. and it is not relevant to the choice of reflections for refinement.The program used for refinement, Jana2006, uses the weighting scheme based on the experimental expectations, see _refine_ls_weighting_details, that does not force *S* to be one. Therefore the values of *S* are usually larger than the ones from the *SHELX* program.

### Fractional atomic coordinates and isotropic or equivalent isotropic displacement parameters (Å^2^)

**Table:**

	*x*	*y*	*z*	*U*_iso_*/*U*_eq_	
P1	0.77023 (2)	0.09776 (3)	0.113631 (14)	0.01755 (11)	
O1	0.93221 (8)	−0.03291 (9)	0.16541 (5)	0.0296 (3)	
O2	0.22566 (11)	0.26725 (9)	0.14746 (5)	0.0446 (4)	
O3	0.30174 (9)	0.15360 (10)	0.20579 (6)	0.0406 (4)	
O4	0.14881 (9)	0.15797 (11)	0.20713 (5)	0.0401 (4)	
N1	0.22509 (10)	0.19384 (10)	0.18695 (5)	0.0268 (4)	
C1	0.83285 (10)	0.21866 (11)	0.13029 (6)	0.0194 (4)	
C2	0.81834 (10)	0.03489 (11)	0.04539 (6)	0.0206 (4)	
C3	0.86270 (11)	−0.04050 (11)	0.19812 (6)	0.0227 (4)	
C4	0.88403 (11)	−0.05841 (13)	−0.06351 (6)	0.0295 (5)	
C5	0.76868 (10)	0.00921 (11)	0.18002 (6)	0.0208 (4)	
C6	0.97263 (12)	0.31195 (13)	0.16513 (8)	0.0333 (5)	
C7	0.59222 (11)	0.16606 (11)	0.14808 (7)	0.0258 (4)	
C8	0.49814 (12)	0.19171 (12)	0.13750 (8)	0.0323 (5)	
C9	0.64745 (10)	0.12851 (11)	0.09836 (6)	0.0214 (4)	
C10	0.79251 (12)	−0.06863 (12)	0.03122 (6)	0.0275 (4)	
C11	0.45787 (12)	0.17953 (13)	0.07799 (9)	0.0361 (5)	
C12	0.91054 (11)	0.04412 (13)	−0.04893 (6)	0.0277 (4)	
C13	0.87750 (11)	0.09150 (12)	0.00565 (6)	0.0229 (4)	
C14	0.83609 (12)	0.40906 (12)	0.12938 (7)	0.0286 (5)	
C15	0.92558 (11)	0.21760 (12)	0.15421 (7)	0.0267 (4)	
C16	0.78818 (11)	0.31533 (11)	0.11762 (6)	0.0231 (4)	
C17	0.51200 (13)	0.14219 (13)	0.02899 (8)	0.0353 (5)	
C18	0.92784 (13)	0.40728 (13)	0.15289 (8)	0.0323 (5)	
C19	0.82556 (13)	−0.11492 (13)	−0.02360 (7)	0.0320 (5)	
C20	0.86235 (13)	−0.09946 (13)	0.25901 (7)	0.0314 (5)	
C21	0.60690 (11)	0.11658 (12)	0.03874 (7)	0.0279 (4)	
H1c4	0.90622	−0.090662	−0.101466	0.0354*	
H1c5	0.7458 (12)	0.0463 (12)	0.2162 (5)	0.025*	
H3c5	0.7248 (10)	−0.0467 (10)	0.1703 (8)	0.025*	
H1c6	1.036543	0.311308	0.18125	0.04*	
H1c7	0.619748	0.173886	0.189322	0.0309*	
H1c8	0.460415	0.218052	0.171378	0.0387*	
H1c10	0.7523	−0.107678	0.058995	0.033*	
H1c11	0.392337	0.197082	0.070905	0.0433*	
H1c12	0.951668	0.082514	−0.076422	0.0333*	
H1c13	0.895387	0.162776	0.015848	0.0275*	
H1c14	0.8054	0.475496	0.121157	0.0343*	
H1c15	0.956584	0.151559	0.163037	0.032*	
H1c16	0.724642	0.316673	0.100864	0.0277*	
H1c17	0.483862	0.133891	−0.012037	0.0424*	
H1c18	0.96073	0.472515	0.160779	0.0387*	
H1c19	0.807881	−0.186217	−0.033893	0.0384*	
H1c20	0.808354	−0.145686	0.260001	0.0377*	
H2c20	0.919458	−0.140447	0.262926	0.0377*	
H3c20	0.85903	−0.050131	0.293224	0.0377*	
H1c21	0.64433	0.090806	0.004569	0.0334*	

### Atomic displacement parameters (Å^2^)

**Table:**

	*U*^11^	*U*^22^	*U*^33^	*U*^12^	*U*^13^	*U*^23^
P1	0.0193 (2)	0.01481 (19)	0.01853 (19)	0.00064 (12)	0.00178 (13)	−0.00047 (11)
O1	0.0281 (6)	0.0311 (6)	0.0297 (5)	0.0062 (4)	0.0058 (4)	0.0063 (4)
O2	0.0793 (10)	0.0237 (6)	0.0305 (6)	−0.0097 (6)	−0.0068 (6)	0.0070 (5)
O3	0.0302 (7)	0.0460 (7)	0.0457 (6)	−0.0026 (6)	0.0044 (5)	0.0129 (5)
O4	0.0328 (7)	0.0494 (8)	0.0381 (6)	0.0049 (6)	0.0030 (5)	0.0117 (5)
N1	0.0398 (8)	0.0197 (6)	0.0210 (6)	−0.0021 (5)	−0.0003 (5)	−0.0017 (4)
C1	0.0231 (7)	0.0170 (7)	0.0183 (6)	−0.0006 (5)	0.0028 (5)	−0.0008 (5)
C2	0.0220 (7)	0.0207 (7)	0.0191 (6)	0.0039 (5)	0.0000 (5)	0.0000 (5)
C3	0.0291 (8)	0.0157 (7)	0.0232 (6)	0.0018 (5)	0.0018 (5)	−0.0002 (5)
C4	0.0296 (8)	0.0387 (9)	0.0200 (6)	0.0109 (7)	−0.0019 (5)	−0.0057 (6)
C5	0.0254 (7)	0.0178 (7)	0.0195 (6)	0.0000 (5)	0.0041 (5)	0.0004 (5)
C6	0.0291 (9)	0.0282 (8)	0.0424 (8)	−0.0048 (7)	−0.0080 (6)	0.0019 (6)
C7	0.0238 (8)	0.0194 (7)	0.0342 (7)	−0.0016 (6)	0.0030 (6)	−0.0029 (6)
C8	0.0248 (8)	0.0221 (8)	0.0502 (9)	−0.0009 (6)	0.0068 (7)	−0.0014 (6)
C9	0.0207 (7)	0.0156 (7)	0.0280 (7)	−0.0028 (5)	−0.0001 (5)	0.0015 (5)
C10	0.0369 (9)	0.0225 (7)	0.0233 (7)	−0.0015 (6)	0.0032 (6)	−0.0020 (6)
C11	0.0211 (8)	0.0266 (8)	0.0604 (10)	−0.0010 (6)	−0.0050 (7)	0.0088 (7)
C12	0.0246 (8)	0.0372 (9)	0.0216 (6)	0.0068 (6)	0.0031 (5)	0.0044 (6)
C13	0.0226 (7)	0.0237 (8)	0.0226 (6)	0.0040 (6)	0.0002 (5)	0.0028 (5)
C14	0.0339 (9)	0.0175 (7)	0.0344 (7)	−0.0005 (6)	0.0013 (6)	0.0012 (6)
C15	0.0257 (8)	0.0209 (7)	0.0334 (7)	0.0011 (6)	−0.0021 (6)	0.0021 (6)
C16	0.0246 (8)	0.0211 (7)	0.0236 (6)	0.0012 (6)	0.0010 (5)	0.0007 (5)
C17	0.0311 (9)	0.0330 (9)	0.0415 (9)	−0.0042 (7)	−0.0112 (7)	0.0095 (7)
C18	0.0363 (9)	0.0219 (8)	0.0385 (8)	−0.0078 (6)	−0.0032 (7)	−0.0008 (6)
C19	0.0422 (10)	0.0263 (8)	0.0274 (7)	0.0034 (7)	−0.0024 (6)	−0.0078 (6)
C20	0.0351 (9)	0.0307 (9)	0.0284 (7)	0.0008 (7)	0.0002 (6)	0.0089 (6)
C21	0.0292 (8)	0.0256 (8)	0.0288 (7)	−0.0017 (6)	−0.0027 (6)	0.0041 (6)

### Geometric parameters (Å, º)

**Table:**

P1—C1	1.7984 (14)	C7—H1c7	0.96
P1—C2	1.7947 (13)	C8—C11	1.390 (2)
P1—C5	1.8024 (13)	C8—H1c8	0.96
P1—C9	1.7992 (15)	C9—C21	1.393 (2)
O1—C3	1.2120 (18)	C10—C19	1.390 (2)
O2—N1	1.2520 (16)	C10—H1c10	0.96
O3—N1	1.2554 (18)	C11—C17	1.382 (2)
O4—N1	1.2475 (18)	C11—H1c11	0.96
C1—C15	1.397 (2)	C12—C13	1.391 (2)
C1—C16	1.400 (2)	C12—H1c12	0.96
C2—C10	1.391 (2)	C13—H1c13	0.96
C2—C13	1.392 (2)	C14—C16	1.385 (2)
C3—C5	1.512 (2)	C14—C18	1.381 (2)
C3—C20	1.494 (2)	C14—H1c14	0.96
C4—C12	1.383 (2)	C15—H1c15	0.96
C4—C19	1.388 (2)	C16—H1c16	0.96
C4—H1c4	0.96	C17—C21	1.389 (2)
C5—H1c5	0.960 (13)	C17—H1c17	0.96
C5—H3c5	0.960 (13)	C18—H1c18	0.96
C6—C15	1.383 (2)	C19—H1c19	0.96
C6—C18	1.384 (2)	C20—H1c20	0.96
C6—H1c6	0.96	C20—H2c20	0.96
C7—C8	1.381 (2)	C20—H3c20	0.96
C7—C9	1.403 (2)	C21—H1c21	0.96
			
C1—P1—C2	110.29 (6)	C2—C10—C19	119.26 (14)
C1—P1—C5	112.59 (6)	C2—C10—H1c10	120.37
C1—P1—C9	108.70 (6)	C19—C10—H1c10	120.37
C2—P1—C5	111.49 (6)	C8—C11—C17	120.10 (16)
C2—P1—C9	108.74 (6)	C8—C11—H1c11	119.95
C5—P1—C9	104.79 (7)	C17—C11—H1c11	119.95
O2—N1—O3	120.16 (14)	C4—C12—C13	119.91 (14)
O2—N1—O4	120.84 (14)	C4—C12—H1c12	120.05
O3—N1—O4	118.99 (12)	C13—C12—H1c12	120.05
P1—C1—C15	121.23 (11)	C2—C13—C12	119.58 (14)
P1—C1—C16	119.07 (11)	C2—C13—H1c13	120.21
C15—C1—C16	119.69 (13)	C12—C13—H1c13	120.21
P1—C2—C10	119.46 (11)	C16—C14—C18	120.24 (14)
P1—C2—C13	119.85 (11)	C16—C14—H1c14	119.88
C10—C2—C13	120.63 (13)	C18—C14—H1c14	119.88
O1—C3—C5	122.18 (12)	C1—C15—C6	119.83 (14)
O1—C3—C20	123.22 (14)	C1—C15—H1c15	120.08
C5—C3—C20	114.60 (12)	C6—C15—H1c15	120.08
C12—C4—C19	120.40 (14)	C1—C16—C14	119.68 (14)
C12—C4—H1c4	119.8	C1—C16—H1c16	120.16
C19—C4—H1c4	119.8	C14—C16—H1c16	120.16
P1—C5—C3	116.05 (10)	C11—C17—C21	120.40 (16)
P1—C5—H1c5	109.4 (8)	C11—C17—H1c17	119.8
P1—C5—H3c5	107.6 (9)	C21—C17—H1c17	119.8
C3—C5—H1c5	107.5 (9)	C6—C18—C14	120.35 (15)
C3—C5—H3c5	108.0 (8)	C6—C18—H1c18	119.82
H1c5—C5—H3c5	108.1 (13)	C14—C18—H1c18	119.82
C15—C6—C18	120.20 (16)	C4—C19—C10	120.22 (15)
C15—C6—H1c6	119.9	C4—C19—H1c19	119.89
C18—C6—H1c6	119.9	C10—C19—H1c19	119.89
C8—C7—C9	119.78 (14)	C3—C20—H1c20	109.47
C8—C7—H1c7	120.11	C3—C20—H2c20	109.47
C9—C7—H1c7	120.11	C3—C20—H3c20	109.47
C7—C8—C11	120.24 (15)	H1c20—C20—H2c20	109.47
C7—C8—H1c8	119.88	H1c20—C20—H3c20	109.47
C11—C8—H1c8	119.88	H2c20—C20—H3c20	109.47
P1—C9—C7	118.54 (11)	C9—C21—C17	119.65 (14)
P1—C9—C21	121.64 (11)	C9—C21—H1c21	120.17
C7—C9—C21	119.83 (14)	C17—C21—H1c21	120.17

### Hydrogen-bond geometry (Å, º)

**Table:**

*D*—H···*A*	*D*—H	H···*A*	*D*···*A*	*D*—H···*A*
C5—H1*c*5···O3^i^	0.960 (13)	2.252 (13)	3.2053 (18)	172.1 (12)
C5—H3*c*5···O2^ii^	0.960 (13)	2.403 (12)	3.1936 (18)	139.4 (11)
C7—H1*c*7···O3^i^	0.96	2.49	3.4365 (19)	167.90
C8—H1*c*8···O3	0.96	2.50	3.177 (2)	127.75
C10—H1*c*10···O2^ii^	0.96	2.49	3.3706 (19)	152.50
C15—H1*c*15···O1	0.96	2.36	3.1780 (19)	142.99

Symmetry codes: (i) −*x*+1, *y*, −*z*+1/2; (ii) *x*+1/2, *y*−1/2, *z*.
